# Cross-talk modulation between ABA and ethylene by transcription factor *SlZFP2* during fruit development and ripening in tomato

**DOI:** 10.1080/15592324.2015.1107691

**Published:** 2015-10-22

**Authors:** Lin Weng, Fangfang Zhao, Rong Li, Han Xiao

**Affiliations:** 1 National Key Laboratory of Plant Molecular Genetics; Institute of Plant Physiology and Ecology; Shanghai Institutes for Biological Sciences; Chinese Academy of Sciences; Shanghai, China

**Keywords:** fruit ripening, overexpression, RNA interference, *S.lycopersicum*, transcription factor

## Abstract

The stress hormone ABA not only regulates stress response, but is also required for plant development and growth. Some evidences indicate that ABA plays a pivotal role in the ripening process of non climacteric as well as climacteric fruits. In a recent study, we showed that the tomato (*Solanum lycopersicum*) transcription factor *SlZFP2* fine tunes ABA biosynthesis during fruit development through direct suppression of ABA biosynthetic genes and it also regulates fruit ripening through transcriptional suppression of the ripening regulator *CNR*. This indicates that *SlZFP2* likely modulates the cross-talk between ABA and ethylene in regulation of fruit development and ripening in tomato. Gene expression analysis using ABA deficient mutants *sit* and *flc* as well as the *SlZFP2* RNAi lines of high fruit ABA production showed that ethylene biosynthetic genes *LeACS1A, LeACS1* and *LeACO1* were positively regulated by ABA during early fruit growth. We reason that ABA promotes basal ethylene biosynthesis in system 1 during fruit growth and likely plays a minor role in ripening regulation after the onset of ripening process.

## Abbreviations

*SlZFP2*: *Solanum lycopersicum zinc finger protein 2*
*HA-SlZFP2*: an expression cassette of hemagglutinin-SlZFP2 fusion protein
ACS: 1-aminocyclopropane-1-carboxylic acid synthase
ACO: ACC oxidase
*CNR*: *COLORLESS NON-RIPENING*
*NOT*: *NOTABILIS*
*SIT*: *SITIENS*
*FLC*: *FLACCA*
*SlAO1*: *Solanum lycopersicum aldehyde oxidase 1*
ABA: abscisic acid
dpa: days post anthesis
RNAi: RNA interference
FPKM: Fragments Per Kilobase of transcript per Million mapped reads.

## 

ABA is well known for its roles in seed maturation and germination, in addition to its pivotal roles in stress response.^1^ At transcriptional level, ABA biosynthesis is regulated by stresses and also developmental processes, for example, during fruit development.^2,3^ Recently, it has been hypothesized that ABA may be involved in ripening regulation of non-climacteric and climacteric fruits.^4-6^ Understanding the transcriptional regulation of ABA biosynthesis during fruit development is required to dissect the role of ABA in regulation of ripening process. In a recent publication, we characterized the role of the transcription factor *SlZFP2*, encoding a single C_2_H_2_ zinc finger protein, in tomato fruit development.^7^ In that study, we found that constitutive expression of *HA-SlZFP2* under 35S promoter repressed ABA biosynthesis in leaves and fruits, whereas silencing its expression increased ABA production in young fruits at 5 and 10 dpa. We also revealed that *SlZFP2* regulates fruit ripening through transcriptional repression of the ripening regulator *CNR*. Thus, the *SlZFP2* pathway likely modulates crosstalk between ABA biosynthesis and the regulatory network of fruit ripening in tomato.

**Figure 1.**
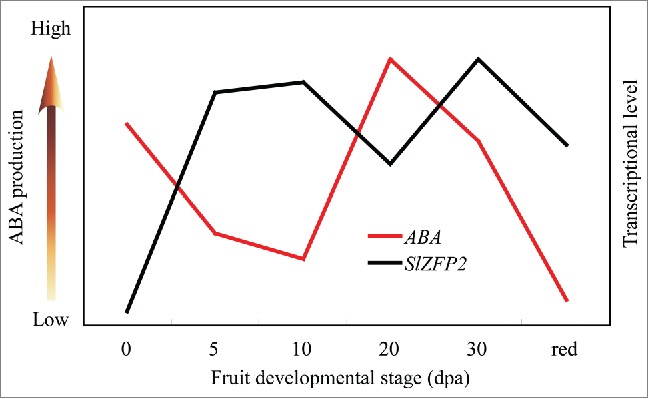
Changes of ABA production and *SlZFP2* expression during fruit development and ripening. The figure was draw from our previous published data in (Weng et al. 2015).^7^ Transcript levels of *SlZFP2* during fruit development and ripening were determined by qRT-PCR, and the maximal levels of *SlZFP2* expression and ABA content were set at 1.

In tomato, there are two ABA peak levels during fruit development and ripening.^2,6^ ABA level is high in anthesis ovaries, and then declines rapidly after pollination.^8,9^ By monitoring the ABA contents in developing fruits, we also found ABA production decreases to relatively low level around 5 dpa, whereas during the cell expansion phase of fruit development ABA production resumes gradually and reaches its second highest level at mature green stage. Through biochemical and gene expression analysis, we have demonstrated that SlZFP2 suppresses ABA biosynthesis through direct binding to the promoters of the ABA biosynthetic genes *NOT, FLC, SIT* and *SlAO1*. Since *SlZFP2* is mainly expressed during fruit development, it likely plays an important role in maintenance of the dynamic ABA production post pollination. Indeed, *SlZFP2* expression negatively correlates with ABA level during fruit development, for example, *SlZFP2* expression was relatively low in anthesis ovaries and 20 dpa fruits when high ABA production was observed (**Fig. 1**). Moreover, we found *SlZFP2* expression was downregulated in the young fruits of ABA deficient mutants *sit* and *flc*, indicating that there is a feedback regulation on *SlZFP2* expression by ABA during fruit development. Similarly, its Arabidopsis homolog *AtZFP2* can be induced by ABA in seedlings.^10^ These results suggest that ABA activates *SlZFP2* and the latter in turn represses ABA biosynthesis during fruit development.

Besides its role in fine tuning ABA biosynthesis during fruit development, our study has also revealed that *SlZFP2* regulates fruit ripening because overexpressing *SlZFP2* or *HA-SlZFP2* delayed fruit ripening for 5–7 days, whereas silencing its expression by RNAi accelerated fruit ripening. Fruit ripening in tomato is mainly mediated by ethylene, which its production is transcriptionally regulated by several transcription factors.^11,12^ Among those ripening regulators, *CNR* inhibits fruit ripening through *AP2a* mediated negative regulation of ethylene biosynthesis and signaling.^13-15^ In *HA-SlZFP2* overexpression and RNAi lines, *CNR* was respectively repressed and upregulated during ripening process, demonstrating that *SlZFP2* regulates ripening process through *CNR* pathway. Since downregulation of *SlZFP2* led to elevated *CNR* expression in fruits as early as 15 dpa, *SlZFP2* likely functions to prevent *CNR* expression before the onset of ripening process.

However, the action of *SlZFP2* on fruit ripening is more likely through indirect impact on ethylene production because overexpression of this transcription factor only resulted in increased expression of ethylene biosynthetic genes *LeACS6, LeACO1* and *LeACO3* in ripe fruits at B10 stage (breaker plus 10 days). Their expression was not impacted at the onset of ripening process by overexpression or RNAi-mediated repression of *SlZFP2*. Thus, the gene expression analysis suggests that elevated or repressed ABA biosynthesis by manipulating *SlZFP2* expression has little impact on ethylene production at the onset of ripening process.

**Figure 2.**
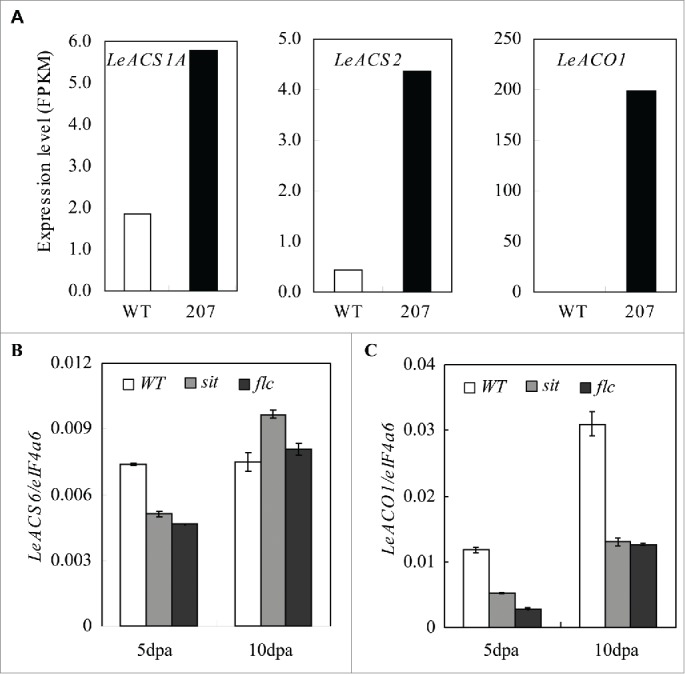
Expression of several ethylene biosynthetic genes in early growth fruits of ABA deficient and overproducing mutants Expression of *LeACS1A, LeACS2* and *LeACO1* (A) was upregulated in the 2 dpa fruits of the *SlZFP2* RNAi line 207, whereas, *LeACS6* (B) and *LeACO1* (C) was downregulated in the 5 and 10 dpa fruits of the ABA deficient mutants *sit* and *flc*. Expression of *LeACS6* and *LeACO1* was determined by qRT-PCR in 3 biological replicates, and the error bars represent standard deviations of the means. The expression values in (A) were from our previous RNA-seq data (Weng et al 2015).^7^

Ethylene is the predominant plant hormone regulating climacteric-fruit ripening. In tomato, two systems of ethylene biosynthesis have been proposed, which basal ethylene production is maintained in system 1 during fruit growth and later its production is increased drastically in system 2 during ripening.^4^ In system 1, *LeACS1A, LeACS6, LeACO1, 3* and *4* are responsible for the basal ethylene production.^4,16-18^ Apparently, *SlZFP2* does not directly regulate the induction of ethylene biosynthesis in system 2. However, we found *LeACO3* and *LeACO4* expression was increased significantly in the 2 dpa fruits of the representative *SlZFP2* RNAi line 207 through transcriptome analysis by RNA-seq. The other ethylene biosynthetic genes *LeACS1A, LeACS2* and *LeACO1*, although the *LeACS1A* and *LeACS2* were expressed at low levels, were also expressed at higher levels in the young fruits (**Fig. 2A**). This suggests that *SlZFP2* may regulate ethylene biosynthesis in system 1. Thus, the problematic fruit set observed in these *SlZFP2* RNAi lines can be explained by elevated ethylene biosynthesis. Our observation is consistent with early studies that ABA promotes flower and fruit abscission through ethylene biosynthesis.^19,20^ Given the significant increase in ABA content in *SlZFP2* RNAi fruits, *SlZFP2* likely regulates ethylene biosynthesis during early fruit growth through ABA pathway. To test the possibility, we analyzed the expression of ethylene biosynthetic genes in ABA deficient mutants *sit* and *flc*. We found that *LeACO1* was downregulated in both the 5 and 10 dpa fruits of the 2 mutants; *LeACS6* expression was also repressed at 5 dpa (**Fig. 2B and C**). The result further supports the role of ABA in promoting ethylene biosynthesis in system 1. Similarly, ABA inhibits root growth also through enhancing ethylene biosynthesis in Arabidopsis.^21^ Therefore, if there is an indispensible role for ABA in regulation of fruit ripening, it will possibly lie on its positive effect on basal ethylene production.

**Figure 3.**
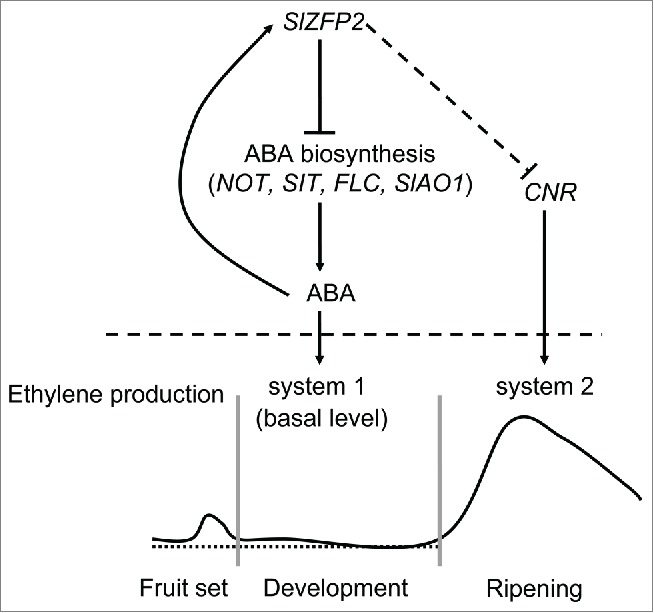
A proposed model for *SlZFP2* action on fruit development and ripening During fruit set and development, *SlZFP2* acts as a transcription repressor to fine tune ABA biosynthesis through direct binding to the promoters of *NOT, SIT, FLC* and *SlAO1*. Decreasing ABA biosynthesis by high *SlZFP2* expression leads to relatively lower ethylene production which facilitates fruit set and prevents floral organ senescence. In addition, *SlZFP2* also prevents the expression of the ripening regulator *CNR* before the onset of ripening process, either directly or indirectly.

Collectively, *SlZFP2* plays at least two roles in regulation of fruit development and ripening (**Fig. 3**). First, *SlZFP2*, likely induced by high ABA at anthesis, represses ABA biosynthesis after anthesis, and in turn the decrease in ABA level limits ethylene production during fruit set and early fruit growth. Fine-tuning ABA biosynthesis likely helps to maintain ethylene production at its basal level in system 1 for normal fruit growth. Second, *SlZFP2* also prevents *CNR* expression before the onset of ripening process. However, it remains to be determined whether or not the ABA biosynthesis regulated by *SlZFP2* interconnects with the *CNR*-mediated ripening regulation.
